# Role of ^18^ F-FDG-PET/CT in an AML-M5a Subtype Patient with Rare Constellation of Hemophagocytic Lymphohistiocytosis & Bilateral Multiple Breast Chloromas

**DOI:** 10.1055/s-0044-1779280

**Published:** 2024-02-13

**Authors:** Yuvan Shrinivas, Shanmuga Sundaram Palaniswamy, Padma Subramanyam

**Affiliations:** 1 Amrita School of Medicine, Amrita Vishwa Vidyapeetham University, Cochin, Kerala, India; 2 Department of Radiodiagnosis, GITAM Institute of Medical Sciences And Research (deemed to be university), Visakhapatnam, Andhra Pradesh, India; 3 Department of Nuclear Medicine & Molecular Imaging, Amrita Institute of Medical Sciences & Research Centre, Cochin, Kerala, India

**Keywords:** AML, hemophagocytic lymphohistiocytosis, fever, whole-body ^18^ F-FDG-PET/CT, chloromas, breast

## Abstract

We report a treated case of acute myeloid leukemia (AML-M5a subtype) with monocytic differentiation (AMoL) presenting with fever and body pains. Initial ^18^ F-FDG-PET/CT ( ^18^ F-flurodeoxyglucose positron emission tomography/computed tomography) identified multiple lymph nodal, and marrow lesions. Biopsy confirmed hemophagocytic lymphohistiocytosis (HLH). Post HLH treatment, follow-up PET/CT demonstrated unsuspected FDG avid bilateral breast lesions ( *n* = 5), which proved to be chloromas, that is, extranodal manifestation of AML. ^18^ F-FDG-PET/CT has helped not only in identifying the various sites of disease involvement but also in guiding the sites for biopsy. Finally, ^18^ F-FDG-PET/CT was useful in monitoring therapy response for both these coexisting pathologies, which are said to be resistant to treatment based on FLT3-ITD tyrosine kinase-3 internal tandem duplication mutation positivity and high-grade AML status. This case represents a rare constellation of different etiologies that needed to be differentiated. It also emphasizes the challenges in interpreting PET/CT findings, especially in difficult clinical scenarios. Disease distribution in HLH/presence of chloromas, etc., can mimic stage IV lymphoma in a known case of AML. So the nuclear medicine physician should be aware of the different complications in the background of AML, especially in patients with poor prognostic factors.

## Introduction

Acute monocytic leukemia (AMoL or AML-M5) is a type of acute myeloid leukemia (AML) with more than 20% blasts in the bone marrow. Of these, more than 80% are of monocytic lineage. The French–American–British (FAB) classification subdivides the AMLs according to the cytomorphological features of peripheral blood and bone marrow cells. AML-M5 is further subclassified into the following: (1) M5a, predominantly monoblasts (> 80%), therefore known as acute monoblastic leukemia and (2) M5b, a mixture of monoblasts and promonocytes (< 80% blasts). AML-M5 has an increased frequency of expression of CD56 and expresses CD34 less frequently, along with 11q23 translocations when compared to other AML subtypes. Acute monoblastic leukemia comprises 5 to 8% of AML cases. It may occur at any age but is most common in young adults. Extramedullary lesions may also occur. AMoL comprises 3 to 6% of cases; the male-to-female ratio is 1.8:1.0. A rare immune-related complication of AML includes hemophagocytic lymphohistiocytosis (HLH), which is a potentially life-threatening syndrome with significant pathological inflammation. It is characterized by an unchecked and persistent activation of cytotoxic T lymphocytes and natural killer (NK) cells. 1 2 Failure to control the immune response leads to increased secretion of inflammatory cytokines and macrophage activation, causing systemic inflammatory symptoms and signs. The immune-mediated injury is directed against multiple organs, and the severity of inflammation distinguishes it from other less serious inflammatory disorders. Clinically, HLH presents a diagnostic challenge because there is no one pathognomonic clinical manifestation or laboratory finding and signs are often nonspecific. FDG-PET/CT may reveal the nodal and other sites of disease involvement.

Another potentially difficult complication of AML is chloroma, 3 also known as myeloid sarcoma. It is a rare extramedullary manifestation (eAML) of myeloid hematological malignancies mostly associated with AML. eAML can easily be misdiagnosed in 40% of cases, especially with lymphomas. Histological confirmation is essential for diagnosis, as well as cytogenetic and molecular testing. The importance of eAML lies in the fact that it requires advanced methods for diagnosis, and identification of targetable mutations, which may be necessary to direct therapeutic approaches. As per the WHO classification, chloromas occur as a result of nodular proliferation of neoplastic cells, characterized by the formation of clinically evident tumors containing immature myeloid cells in the extramedullary sites. Most common sites of involvement include the skin, soft tissues, the central nervous system (CNS), and the urogenital tract. Breast chloromas are rare and thought to be resistant to treatment. AML-like induction chemotherapy regimens remain the standard of care, and consolidation therapy is controversial. Failure to achieve complete remission is associated with worse survival outcomes. Allogeneic hematopoietic transplant may be considered in first complete remission. Posttransplant relapses are common and responsible for increased mortality and shorter survival.

We report a young lady who is a treated case of AML (M5a subtype) with monocytic differentiation (AMoL) presenting with fever and body pains. FDG-PET/CT identified multiple lymph nodal and marrow lesions. Biopsy confirmed HLH. Within 2 months of HLH detection and treatment, follow-up PET/CT demonstrated unsuspected bilateral breast lesions, which proved to be chloromas, that is, extranodal manifestation of AML.

## Case Report

A 30-year-old lady was diagnosed with AML in June 2021. Bone marrow aspiration reported high-grade hematopoietic neoplasm, and bone marrow biopsy described variably cellular marrow with diffuse infiltration by large plasmacytoid cells. Flow cytometry studies revealed AML with monocytic differentiation. Karyotyping was also performed. Genomic investigations of AML were carried out. The patient completed treatment in December 2021. After 3 months of symptom-free period, the patient presented with prolonged fever for 3 weeks and body pains.

**Fig. 1:**
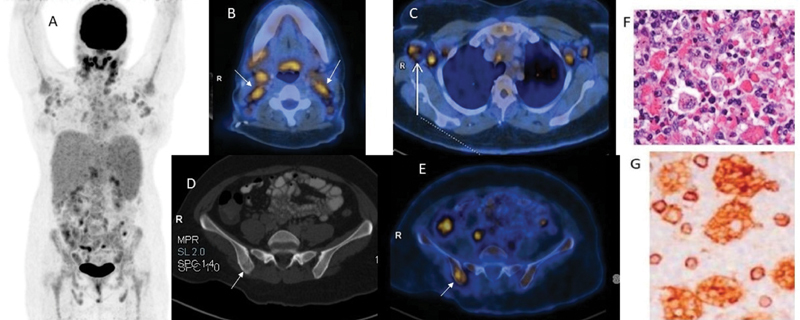
( **A** ) Positron emission tomography/computed tomography (PE/TCT; maximum intensity projection [MIP]) revealed nodal ( **B:** bilateral cervical ( *arrow* ); **C:** axillary) and extranodal lesions, confirming secondary hemophagocytic lymphohistiocytosis (HLH) on biopsy. ( **D,E** ) Transaxial CT and fused PET-CT images reveal Fluorodeoxyglucose (FDG) avid lytic right iliac bone and marrow involvement ( *arrow* ). Biopsy from left axillary lymph node showed sinuses are dilated and filled with benign-appearing histiocytes on hematoxylin and eosin (H&E) stain. There is erythrophagocytosis by macrophages ( **F** ) highlighted using histiocytic markers (CD4 in **G** ).

FDG-PET/CT revealed cervical, axillary lymph nodal, and lytic right iliac bone and marrow involvement, with gross hepatosplenomegaly ( Fig. 1 ). Biopsy confirmed secondary HLH. The diagnostic criteria for HLH are fever (peak temperature of > 38.5°C for > 7 days), splenomegaly (spleen palpable > 3 cm below the costal margin), cytopenia involving greater than two cell lines (hemoglobin < 9 g/dL [90 g/L], absolute neutrophil count < 100/µL [0.10 × 10 ^9^ /L], platelets < 100,000/µL [100 × 10 ^9^ /L]) with raised C-reactive protein (CRP; 72 mg/dL), erythrocyte sedimentation rate (ESR; 50 mm/h), serum ferritin (14,946 ng/mL), and very high lactate dehydrogenase levels (LDH; 11,251 U/L). S Fibrinogen was low (2.4 g/L) with elevated D-dimer (7,500 µg/L). Biopsy from the left axillary lymph node showed the sinuses are dilated and filled with benign-appearing histiocytes on hematoxylin and eosin (H&E) stain ( Fig. 1F ). There is erythrophagocytosis by macrophages. Markers for myeloid cells were found to be positive for CD 117 as seen in Fig. 1G and for CD 68 ( Fig. 1H ). Immunostaining for Myeloperoxidase (MPO), as seen in Fig. 1I was also positive. With these findings on histology, the patient was confirmed to have HLH. The patient was treated with oral dexamethasone 16 mg once a day, tablet cyclosporine 100 mg twice a day, decitabine (20 mg/m ^2^ ; 32 mg for 10 days), midostaurin (type III tyrosine kinase inhibitor), and venetoclax (B-cell lymphoma 2 [BCL2] inhibitor) each for 14 days.

**Fig. 2:**
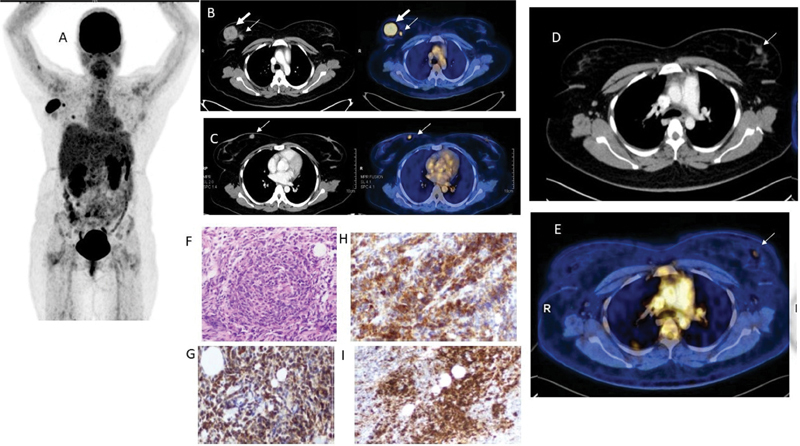
Follow-up FDG-PET/CT (fluorodeoxyglucose positron emission tomography/computed tomography) after 2 months showed complete resolution of all the hemophagocytic lymphohistiocytosis (HLH) lesions. New FDG avid multiple bilateral breast lesions (3 on right, 2 on left) were noted, ( **A** ) MIP image, ( **B** ) and ( **C** ) CECT & fused PETCT images showing right breast a dominant & smaller lesion, with the largest in the upper outer quadrant with SUVmax of 5.6, tiny 2nd lesion tagging inferiorly to dominant nodule. Findings raise a suspicion for new primary breast malignancy or lymphomatous deposits. Biopsy proved to be extramedullary relapse of acute myeloid leukemia (AML) in the form of bilateral breast chloromas. ( **D** ) and ( **E** ) CECT & fused PETCT image showing FDG avid tiny nodule in left breast ( *arrow* ). ( **F** ) Hematoxylin Eosin staining revealed dense myeloid precursor proliferation with breast tissue invasion. ( **G** ) CD117 and ( **H** ) CD 68, which are markers for myeloid cells ( **I** ) Immunostaining positivity for Myeloperoxidase (MPO).

Follow-up FDG-PET/CT after 2 months showed complete resolution of all the HLH lesions. Apart from that, the patient showed new FDG avid multiple bilateral breast lesions (3 on right, 2 on left) ( Fig. 2A ), raising the suspicion for new primary breast malignancy or extranodal lymphomatous deposits given the background of AML ( Fig. 2B–E ). Biopsy from the larger right breast lesion proved to be an extramedullary relapse of AML in the form of bilateral breast chloromas. As they are composed of malignant myeloid precursor cells, the breast lesions reveal a dense myeloid precursor proliferation with breast tissue invasion ( Fig. 2F ; H&E stain). Immunostaining shows positivity for CD117 ( Fig. 2G ), CD68 ( Fig. 2H ), myeloperoxidase (MPO; Fig. 2I ), which are markers of myeloid cells. Molecular analysis showed positivity for FLT3–ITD (Tyrosine kinase-3 internal tandem duplication) mutation. 4 Studies reveal that presence of chloroma is often associated with a poor prognosis, and therefore optimization of treatment is essential. 5

**Fig. 3:**
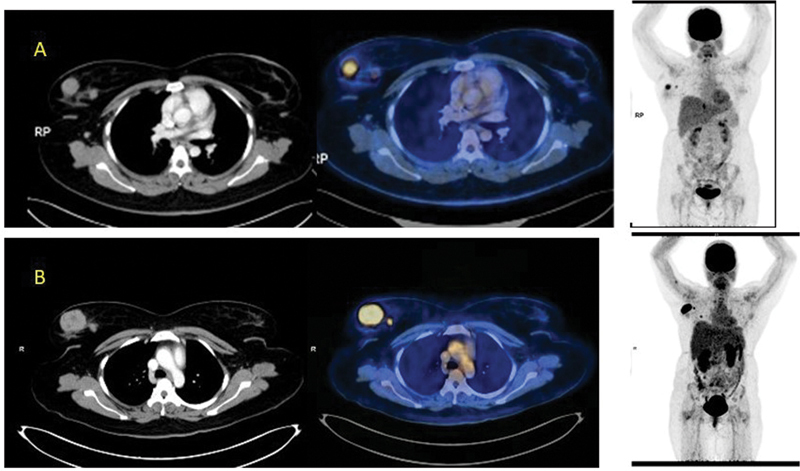
( **A** ) Contrast-enhanced computed tomography (CECT), fused positron emission tomography CT (PET/CT) and maximum intensity projection (MIP) images of previous PET/CT. ( **B** ) CECT, fused PET/CT, and MIP images of postchemotherapy PET/CT. The patient received three cycles of chemotherapy with intermediate-dose cytarabine (INDAC; 3 g/m ^2^ ). Follow-up FDG-PET/CT 2 months later showed resolution of two breast lesions, one each in the right and left breasts. Out of five bilateral breast lesions, only three persisted in spite of treatment (2 on the right breast and 1 on the left breast). Residual lesions in right breast showed an increase in size and FDG avidity, now measuring 4.1 × 3 cm, situated in the right breast upper outer quadrant (previous size was 3.4 × 2.0 cm). The SUVmax of the largest right breast lesion is now 10.2, while the previous SUVmax was 5.6. The smaller right breast residual lesion measures 0.8 cm in size, with the recent SUVmax being 3.4 (previous SUVmax was 2.3). Based on the FDG-PET/CT findings, the patient is being planned for radiotherapy to the residual FDG avid right breast chloromas.

The patient received three cycles of chemotherapy with intermediate-dose cytarabine (INDAC; 3 g/m ^2^ ). Follow-up FDG-PET/CT 2 months later showed persistent well-defined rounded, soft-tissue density lesions in bilateral breasts with increase in size and FDG avidity (2 in the right and 1 in the left with resolution of 2 other breast lesions). The largest lesion measured 4.1 × 3 cm in the right breast upper outer quadrant ( Fig 3 ). SUVmax of the larger right breast lesion was 10.2, which was previously 5.6. There was complete resolution of one lesion each in the right and left breasts. The anatomical and functional status of the chloromas could not have been ascertained without follow-up PET/CT. Based on the PET findings, radiotherapy is being planned for the refractory (persistent FDG avid) right breast chloroma.

## Discussion

This subtype of AML (M5a) is reported to display increasing extramedullary lesions and is said to exhibit an overall poor prognosis. The bone marrow is usually hypercellular, showing a predominant population of large (up to 30 μm in diameter), poorly differentiated blasts with a rounded to oval nucleus containing reticular and immature chromatin pattern and one to four light-blue nucleoli. The cytoplasm is usually abundant and basophilic, with rare scattered azurophilic granules, fine vacuolizations, and absence of Auer rods. By performing FDG-PET/CT, lesions that are clinically occult or not discernible on conventional imaging can be investigated. It also helps in guiding sites for biopsy and tailoring treatment. Multifocal involvement is also found to carry poor prognosis. Studies have shown that FLT3 mutations in AML maybe refractory. Our case reveals FLT3–ITD mutation positivity. In literature survey, *FLT3* occurs in approximately 30% of all AML cases, with internal tandem duplication (ITD) representing the most common type of *FLT3* mutation ( *FLT3* -ITD; ∼25% of all AML cases). It has also been shown that *FLT3* -ITD is a common driver mutation that presents with a high leukemic burden and confers a poor prognosis in patients with AML. 1

HLH is an immune-related etiology, initially named histiocytic medullary reticulosis, and first reported in the literature in 1939 by Scott and Robb-Smith. 2 Initially, this disease was initially thought to involve only pediatric population, but recent studies show that 40% of adults can also get affected with HLH. It is most often triggered by a combination of underlying genetics and acquired exposures. 3 4 Secondary or acquired HLH may result from a malignant, infectious, or autoimmune stimulus in the absence of an identifiable underlying genetic trigger.

Chloromas are a different entity; synonyms are granulocytic sarcoma or myeloid sarcoma. They are greenish-colored tumors ( *chloros* in Greek), composed of malignant myeloid precursor cells. 6 Common sites of chloromas are skin, lymph nodes, soft tissues, bones, CNS, and urogenital tract. 7 Breast involvement is extremely rare, especially as an isolated disease. They occur as painless breast mass. 8 Chloromas occur in 1.2 to 9% of AML patients 5 and have never been reported along with HLH. The presence of chloroma is often associated with a poor prognosis, and therefore optimization of treatment is essential. 9

Our case represents a rare constellation of different etiologies that needed to be differentiated. FDG-PET/CT helped not only in identifying the various sites of disease involvement but also in guiding the sites for biopsy. Our AML patient had two different disease entities in the background of AML requiring different management strategies. Finally, FDG-PET/CT was useful in monitoring therapy response for both these coexisting pathologies, which are said to be resistant to treatment based on FLT3–ITD mutation positivity and high-grade AML status. This case emphasizes the challenges in interpreting PET/CT findings, especially in difficult clinical scenarios. Disease distribution in HLH/presence of chloromas, etc., can mimic stage IV lymphoma in a known case of AML. So the nuclear medicine physician should be aware of the different complications in the background of AML, especially in patients with poor prognostic factors.

## Conclusion

The outcome in patients with eAML is variable and depends on several factors including cytogenetic risk profile, isolated or with concurrent marrow involvement, and the presence of any targetable mutations in the extramedullary site or marrow samples. This case emphasizes the incremental role of FDG PETCT in diagnosis and management of both benign and malignant pathologies that can arise in immunocompromised patients. It also provides information in refractory cases where management changes maybe needed based on PETCT findings. Cytogenetic, molecular studies, and PET/CT imaging therefore play a major role in achieving complete remission in such cases.
